# Effects of the combined extracts of Herba Epimedii and Fructus Ligustri Lucidi on bone mineral content and bone turnover in osteoporotic rats

**DOI:** 10.1186/s12906-015-0641-4

**Published:** 2015-04-09

**Authors:** Ren-Hui Liu, Xue Kang, Li-Ping Xu, Hong-Lei Nian, Xin-Wei Yang, Hao-Tian Shi, Xiu-Juan Wang

**Affiliations:** Beijing Key Laboratory of Traditional Chinese Medicine Collateral Disease Theory Research, School of Traditional Chinese Medicine, Capital Medical University, No. 10 Xitoutiao, Youanmenwai, Beijing, Fengtai District 100069 China; Dispensary of Traditional Chinese Medicine, Beijing Jishuitan Hospital, No. 10 Xinjiekou East Street, Beijing, Xicheng District 100035 China

**Keywords:** Herba Epimedii extract, Fructus Ligustri Lucidi extract, Bone biomechanical properties, Bone turnover, Bone mineral content

## Abstract

**Background:**

The decoction combination of Herba Epimedii and Fructus Ligustri Lucidi has been used to treat osteoporosis for almost 50 years by practitioners of traditional Chinese medicine. However, it is unclear what specific effects this combination of herbs has on the skeleton. The aim of this study was to assess the effects of the combined extracts from Herba Epimedii and Fructus Ligustri Lucidi on the bone turnover and bone mineral content in a rat model of osteoporosis induced by retinoic acid.

**Methods:**

Fifty male Wistar rats were randomly assigned to the normal control group, osteoporosis model group, or treatment groups in which osteoporosis was induced and then the combined extracts of Herba Epimedii and Fructus Ligustri Lucidi were administered at 50, 100, or 200 mg/kg/day for 3 weeks via oral gavage. The rat osteoporosis model was induced by intragastric administration of 70 mg/kg/day of retinoic acid for 2 weeks. Bone turnover markers, bone biomechanical properties, and the calcium and phosphorus content of the right tibia and serum were measured.

**Results:**

The retinoic acid administration decreased the bone mass and the contents of calcium and phosphorus in the bone mineral, weakened the biomechanical properties, and increased bone turnover by stimulating bone resorption and collagen metabolism. Treatment with the combined extracts of Herba Epimedii and Fructus Ligustri Lucidi significantly mitigated the effects of osteoporosis on the rats by decreasing bone metabolism, improving the bone mineral content, and increasing the biomechanical properties.

**Conclusions:**

The results of this study highlight the anti-osteoporosis effects of the combined extracts of Herba Epimedii and Fructus Ligustri Lucidi. These findings may contribute to the development of natural anti-osteoporosis herbal medicines.

## Background

Osteoporosis is one of the most common bone remodeling diseases characterized by a reduction in bone mass and bone microstructural deterioration [1]. This disease is caused by an imbalance in bone remodeling, with decreased bone formation and increased bone resorption, which ultimately results in skeletal fragility and an increased risk of hip and vertebral fracture [2]. Osteoporosis mainly affects post-menopausal women and elderly men and is one of the most serious threats to public health [3,4], with an estimated prevalence of 200 million people worldwide and annual costs exceeding approximately 10 billion dollars [5]. Western medical treatments for osteoporosis are somewhat effective, but the long-term use of those medicines can result in a number of side effects [6-9], including atraumatic bone fractures caused by bisphosphonates, increased incidence of coronary heart disease, stroke, pulmonary embolism, and ovarian and endometrial cancers caused by estrogen or estrogen replacement therapy.

Because of these side effects of the currently available anti-osteoporosis drugs, the development of herbal medicinal products to treat osteoporosis has become a global focus, and many studies of natural medicines have been published [10]. According to the theories of traditional Chinese medicine (TCM), bone health is considered to be closely related to kidney function, and “kidney deficiency” is thought to be the root of all pathologies related to bones and joints. Thus, the treatment of osteoporosis is achieved by strengthening the kidney function. The ancient Chinese medicinal literature indicates that Herba Epimedii (*Yinyanghuo*) replenishes kidney-*yang* and Fructus Ligustri Lucidi (*Nvzhenzi*) replenishes kidney-*yin*. Therefore, they have been used to strengthen bone and treat osteoporosis in China for several thousand years. Osteoporosis is a chronic and complex disease related to kidney-deficiency syndrome that should be treated with a long-term medical intervention, but both *Yang*-tonifying prescriptions, which have the property of warm dryness, and *yin*-tonifying prescriptions, which show sticky and greasy properties, are unsuitable for long-term clinical application. However, *Yang*-tonifying herbs matched with the right *Yin*-tonifying herbs can make living things grow freely and flourish by eliminating the side effects of each other. Professor *Shi-Zeng Li*, a famous doctor of TCM, has used the decoction combination of Herba Epimedii and Fructus Ligustri Lucidi to treat osteoporosis for almost 50 years [11]. We previously demonstrated that decoction of Herba Epimedii and Fructus Ligustri Lucidi reversed abnormalities in collagen type I and bone metabolism, increased bone mineral density (BMD) and bone biomechanical properties, and improved the pathological condition of the bone tissue in rats with osteoporosis induced by retinoic acid administration. In addition, the effects of combined therapy with Herba Epimedii and Fructus Ligustri Lucidi were better than with either one alone. Many studies confirming the anti-osteoporotic effects of the total flavonoids of Herba Epimedii [12-14] and the total iridoids and flavonoids extracted from Fructus Ligustri Lucidi [15-17] have been published.

Based on the work referenced above, the aim of this study was to assess the anti-osteoporosis effects of combined extracts of Herba Epimedii and Fructus Ligustri Lucidi on the retinoic acid-induced rat osteoporosis model by measuring bone turnover markers, bone biomechanical properties, and bone mineral content after treatment.

## Methods

### Preparation of herbal extracts

Herba Epimedii, the dried leaf of *Epimedium brevicornum Maxim*, and Fructus Ligustri Lucidi, the dried mature seed of *Ligustrum lucidum Ait*, were purchased from Beijing *Tongrentang* Pharmaceutical Co. Ltd., China, and authenticated by an expert herbalist at Capital Medical University. The plant matter was stored in a dry and sealed container at 4°C.

Herba Epimedii (1000 g) was extracted three times with 90% ethanol (10 L) for 3, 2, and 2 hours at 70°C in a reflux apparatus. The extracts were mixed, filtered, and concentrated under reduced pressure until recovery showed no alcohol precipitation. Next, they were extracted with petroleum ether three times to remove any chlorophyll and then washed with D-101 macroporous resin until becoming colorless, eluted with 90% ethanol, and steamed to yield a dark yellow powder containing the total flavonoids of Herba Epimedii (TFE). The yield of TFE was 2.5%. Based on the phytochemical test (Pharmacopeia of the People’s Republic of China, 2010 Edition), the content of TFE was 80%, calculated using *Icariin* as a standard.

Fructus Ligustri Lucidi (1000 g) was extracted three times with 75% ethanol (10 L) for 3, 2, and 2 hours at 70°C in a reflux apparatus. The extracts were mixed, filtered, and concentrated under reduced pressure until recovery showed no alcohol precipitation. Using AB-8 macroporous resin, the extracts were washed with distilled water and then with 75% ethanol. The steamed extracts included the total iridoids and flavonoids of Fructus Ligustri Lucidi (TIFL), which appeared as a dark yellow powder. The yield of TIFL was 5%. Based on the phytochemical test (Pharmacopeia of the People’s Republic of China, 2010 Edition), the content of TIFL was greater than 80%, as calculated using *Oleanolic Acid* and *Rutin* as standards [11].

The Herba Epimedii and Fructus Ligustri Lucidi extracts were combined at a ratio of 2 to 3 (TFE to TIFL), equivalent to a ratio of 4 to 3 of the raw herbs. Doses of Herba Epimedii and Fructus Ligustri Lucidi were chosen based on the clinical practice by *Shi-Zeng Li* (21 g/kg in human body) and our previous studies (3.5 g/kg in rats), which have demonstrated effective prevention and treatment of osteoporosis. Before application, the TFE and TIFL mixtures were dissolved in deionized water at concentrations of 5, 10, and 20 mg/mL.

### Animals

Fifty male Wistar rats weighing 230–270 g with an average age of 3 months were purchased from Vital River Laboratory Animal Technology Co. Ltd. (Beijing, China) and cared for in the Experimental Animal Center of Capital Medical University. The experimental protocol was approved by the Animal Research Committee of Capital Medical University. During the entire experiment, the animals were housed in stainless cages (three rats per cage) under conventional controlled conditions (temperature: 23 ± 2°C, relative humidity: 50 ± 10%, and 12-hour light–dark cycle). They were allowed free access to the standard laboratory food and tap water.

### Experimental protocol

After acclimatization for 1 week, the rats were randomly divided into five groups of 10 rats each, including a normal control group, osteoporosis model group, and low (Extract-L, 50 mg/kg/day), medium (Extract-M, 100 mg/kg/day), and high (Extract-H, 200 mg/kg/day) treatment groups. All rats except those in the normal control group received intragastric administration of retinoic acid (70 mg/kg) daily for 2 weeks to induce the osteoporosis model. The rats in the treatment groups were administered the combined extracts via oral gavage at the indicated doses. The rats in the normal control group and osteoporosis model group were given oral gavage with the same volume of distilled water. Three weeks later, the rats were anesthetized with 25% ethyl carbamate (4 mL/kg, i.p.). Blood samples were collected and separated using a centrifuge (Biofuge 15R, Heraeus Sepatech, Baxter International, Deerfield, IL, USA). Serum was collected and stored at −80°C until detection of bone turnover markers, calcium, and phosphorus. The right femur and tibia were isolated, wrapped in gauze soaked in 0.9% NaCl solution, and stored at −20°C.

### Serum markers of bone turnover

The serum levels of bone turnover markers, including osteocalcin, alkaline phosphatase (ALP), tartrate-resistant acid phosphatase (TRACP), procollagen type I carboxy-terminal propeptide (PICP), procollagen type I amino-terminal propeptide (PINP), and carboxy-terminal cross linked telopeptide of type I collagen (CTX), were determined. Serum osteocalcin was measured in duplicate using an osteocalcin radioimmunoassay kit (Beijing Sino-UK Institute of Biological Technology, Beijing, China). Serum ALP level was assayed using a commercial kit (Nanjing Jiancheng Bioengineering Institute, Nanjing, China) with a spectrophotometer (Shimadzu, Kyoto, Japan). Serum TRACP, PICP, PINP, and CTX were measured by enzyme-linked immunosorbent assays (Immunodiagnostic Systems Ltd., Boldon, UK) with the absorbance read on an ELISA reader (Thermo, Waltham, MA, USA) at 450 nm.

### Bone biomechanical testing

The biomechanical properties of the right femur were assessed using a diaphysis bending test [18] on a WD-1 Universal Testing Apparatus (KeXin Testing Machine Co., Ltd., Changchun, China) that was equipped with calculation and analysis software. The two ends of the right femur were horizontally fixed on the three-point bending platform, and a vertical compression load was then applied to the mid-point of the femur at a displacement rate of 2 mm/min. The Loading-displacement curves were recorded on-line and analyzed to determing the bone biomechanical properties of ultimate load (the force at which fracture occurs) and displacement at ultimat (bone deformation at the fracture point)..

### Bone mineral content

Serum levels of calcium and phosphorus were determined by colorimetric analysis using a calcium reagent set and a phosphorus reagent set (Nanjing Jiancheng Bioengineering Institute) with a spectrophotometer (Shimadzu).

To determine the mass of bone mineral, the right tibia sample was weighed (wet weight), and then dried at 640°C for 12 hours in a muffle furnace. The dried sample was ground into powder and weighed (dry weight). The ratio of the bone mineral mass to the bone mass was determined. One hundred milligrams of bone powder was then dissolved in 6 M HCl and diluted with distilled water. The calcium and phosphorus contents were measured using atomic absorption spectrophotometry (Analyst 100 Spectrophotometer, PerkinElmer, Waltham, MA, USA).

### Statistical analysis

Data are presented as means ± standard deviation (SD). The data analysis was performed in SPSS 13.0 (SPSS Inc., Chicago, IL, USA). All of the data were tested for normality using the Kolmogorov–Smirnov test and passed. Differences between the groups were tested using analysis of variance followed by Duncan’s multiple range post-hoc test. *P-*values less than 0.05 were considered statistically significant.

## Results

### Serum markers of bone turnover

**Figure 1:**
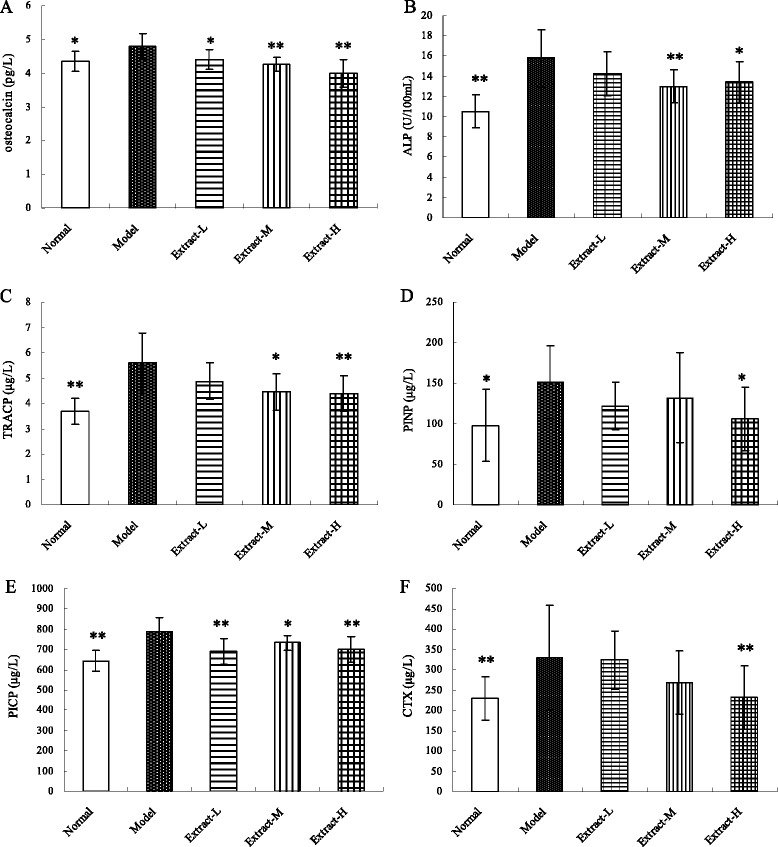
**Effects of the combination with TFE and TIFL on osteocalcin (A), ALP (B), TRACP (C), PICP (D), PINP (E) and CTX (F) in osteoporosis rats.** serum markers of bone turnover, including osteocalcin, ALP, TRACP, PICP, PINP and CTX, all of which were significantly greater in the osteoporosis model group than those in the normal control group. Mean ± SD, *n* = 10. ^*^ *P* < 0.05 and ^**^ *P* < 0.01 *vs* osteoporosis model group.

The levels of serum markers of bone turnover, including osteocalcin, ALP, TRACP, PICP, PINP, and CTX, all of which were significantly higher in the osteoporosis model group than in the normal control group, are shown in Figure 1. Treatment with the combination of TFE and TIFL significantly decreased the osteocalcin and PICP contents compared with those of the osteoporosis group. In addition, the concentrations of ALP and TRACP were significantly decreased in the Extract-M and Extract-H groups, while the levels of PINP and CTX were decreased only in the Extract-H group.

### Bone biomechanical properties

**Figure 2:**
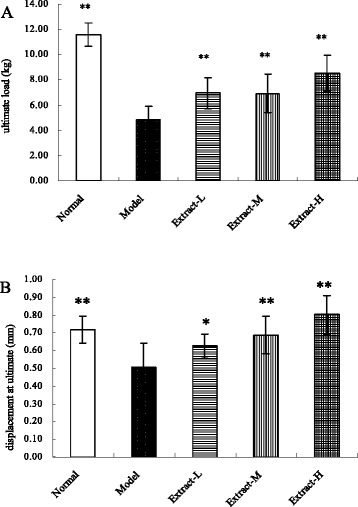
**Effects of the combination with TFE and TIFL on ultimate load (A) and displacement at ultimate (B) of femur biomechanical properties in osteoporosis rats.** Femur ultimate load and displacement at ultimate were significantly decreased in the osteoporosis model group, compared to the normal control group. Mean ± SD, *n* = 10. ^*^ *P* < 0.05 and ^**^ *P* < 0.01 vs osteoporosis model group.

The results of biomechanical testing are shown in Figure 2. The femur ultimate load and displacement at ultimate were significantly lower in the osteoporosis model than in the normal control group. Significant differences in the biomechanical properties of the right femur were found between the osteoporosis group and each of the groups treated with the TFE and TIFL combination.

### Bone mineral content

**Figure 3:**
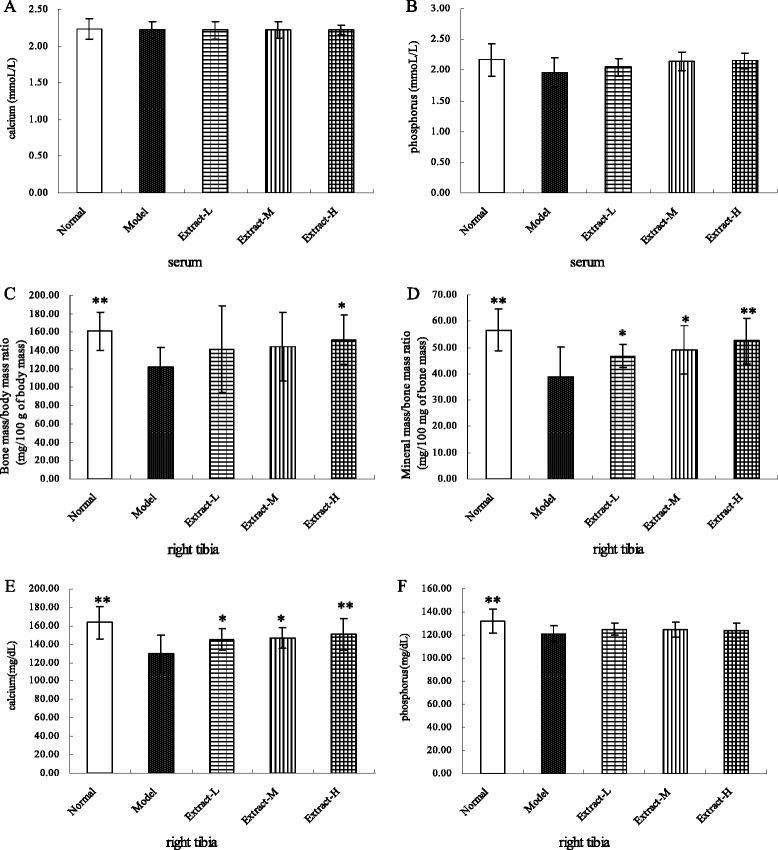
**Effects of the combination with TFE and TIFL on bone mineral contents in osteoporosis rats, including serum calcium (A), serum phosphorus (B), the ratio of bone mass to body mass (C), the ratio of mineral mass to bone mass (D), bone calcium (E) and bone phosphorus (F).** Bone mass/body mass ratio, mineral mass/bone mass ratio, bone calcium and bone phosphorus in the osteoporosis model group were significantly lower than those in the normal group. Mean ± SD, *n* = 10. ^*^ *P* < 0.05 and ^**^ *P* < 0.01 *vs* osteoporosis model group.

The bone mineral contents are shown in Figure 3. No effects of the combined TFE and TIFL treatment on the serum levels of calcium and phosphorus were found. The comparative bone mass (the ratio of bone mass to body mass), comparative mineral mass (the ratio of mineral mass to bone mass),, bone calcium, and bone phosphorus in the osteoporotic rats were significantly lower than those in the normal group (all *P* < 0.01). Treatment with the TFE and TIFL combination at all three doses significantly increased the comparative mineral mass and calcium content in the right tibia compared to those in the osteoporotic rats. In addition, the 200 mg/kg dose increased the comparative bone mass compared to that in the osteoporosis model group.

## Discussion

The kidney-tonifying Chinese herbal medicines Herba Epimedii and Fructus Ligustri Lucidi have been widely used to treat bone disease for thousands of years in China. Professor *Shi-Zeng Li*, a famous doctor of TCM, has used the decoction composed of Herba Epimedii (as a kidney-*Yang* tonifying herb) and Fructus Ligustri Lucidi (as a kidney-*Yin* tonifying herb) to treat osteoporosis for almost 50 years. Preliminary studies from our group confirmed that the decoction and combined extracts of Herba Epimedii and Fructus Ligustri Lucidi showed anti-osteoporotic effects on retinoic acid-induced model of osteoporosis in rats [11]. In fact, the effect of the combined Herba Epimedii and Fructus Ligustri Lucidi therapy was significantly better than either one alone, suggesting that the combination of the two herbs may be good for the prevention and treatment of osteoporosis. These two herbal medicines will undoubtedly continue to be used as a cost-effective alternative to commercial pharmaceuticals for the treatment of osteoporosis by doctors of TCM. However, the effects of the combined extracts of the two herbs for managing osteoporosis have not yet been fully reported. Based on several previous studies elucidating the active ingredients of Herba Epimedii and Fructus Ligustri Lucidi, we chose TFE and TIFL from among the active ingredients of the two herbs in this study for further evaluation of their anti-osteoporosis effects.

Biochemical markers of bone turnover provide important indications for the early diagnosis of osteoporosis and reflect bone formation and resorption through the bone turnover rate [19]. ALP and osteocalcin are biochemical markers of osteogenesis [20,21], while TRACP is a marker of bone resorption [22,23]. Retinoic acid-induced osteoporosis model is a high bone turnover type of osteoporosis characterized by large increases in bone formation and bone resorption. However, the increase in bone resorption is larger than that of bone formation, leading to significant changes in bone turnover resulting in a net bone loss [24]. The higher levels of ALP, TRACP, and osteocalcin in the osteoporosis model group reflect the increase in bone formation that accompanies the excessive increase in bone resorption that normally appears in high conversion-type osteoporosis. After administration of the combined TFE and TIFL for 3 weeks, the levels of ALP, TRACP, and osteocalcin were lower than those of the osteoporosis model group. This suggests that the extracts of the two herbs may help prevent osteoporosis by mitigating the high turnover rate of osteoporotic bone metabolism.

Type I collagen is the major protein product of proliferating osteoblastic cells and comprises more than 90% of the organic matrix of mineralized bone. The higher concentrations of the formation and degradation markers of type I collagen suggest “catch-up growth” of bone collagen matrix and normalization of the bone collagen turnover [25]. PINP and PICP are markers of the formation of the organic matrix of bone and type I collagen synthesis [26]. Serum PINP is reported to be more sensitive than serum PICP to changes in bone turnover [27], but CTX, a marker of type I collagen degradation, is currently the most sensitive marker for bone resorption and bone collagen degradation [28,29]. The results of the present study show that the serum PINP, PICP, and CTX levels in the osteoporosis model group were significantly larger than those in the normal control group, while the combined extracts of Herba Epimedii and Fructus Ligustri Lucidi significantly decreased the serum levels of PINP, PICP, and CTX. These results suggest that the active ingredients in the two herbs may effectively regulate collagen metabolism and bone turnover in osteoporotic rats.

Osteoporosis causes incremental bone fragility, increased fracture risk, and decreased bone mass [30-32]. After treatment with the combined TFE and TIFL, increases in the bone mechanical properties, including ultimate load and displacement at ultimate, were measured. These results indicate the combined extracts of the two herbs could improve the bone biomechanical features, and decrease the fracture risk. The primary change in bone mass during osteoporosis is an increase in the loss of calcium from the skeleton. Because of the rapid bone loss, the secretion of parathyroid hormone decreases, which in turn decreases calcium absorption in the intestine, finally resulting in the loss of calcium from the bone and body [33,34]. In the present experiments, treatment with the combined TFE and TIFL decreased the progression of osteoporosis, as assessed by the bone calcium content and mineral mass. We speculate that the increase in the bone calcium content may have been caused by the high intake of sugars that are present in the combined extracts. Some of the sugars may have reached the gastrointestinal tract and been fermented by the intestinal bacteria, reducing the pH of the environment and increasing calcium absorption. Such a mechanism was proposed for inulin-type fructans, which were reported to increase calcium absorption [35]. This increased calcium absorption was accompanied by an increased mineral mass in the bones of rats fed a diet containing fructooligosaccharides [36].

The detailed compositions of both Herba Epimedii and Fructus Ligustri Lucidi have been previously reported, and mainly include flavonoids, iridoid glycosides, and alkaloids, all of which may contribute to the anti-osteoporotic effects of the two herbs. The total flavonoids from Herba Epimedii are reported to protect against bone loss, including by maintaining the calcium metabolism balance [14], promoting the proliferation of osteoblasts, and suppressing bone resorption [13]. Icariin, Icariside II, and Baohuoside from Herba Epimedii significantly inhibited the proliferation of pre-osteoclast RAW 264.7 cells [13], and synergistic inhibition activity of Icariside II and Icaritin was found [37]. Similarly, the total iridoids and flavonoids from Fructus Ligustri Lucidi showed therapeutic effects on the BMD and absorption of intestinal calcium [15-17]. The ethanol extracts of Fructus Ligustri Lucidi are able to modulate the turnover of bone, calcium balance, and bone properties, as well as enhance the mineralization process [38,39]. These results suggested that the anti-osteoporotic effects of the combined extracts of Epimedii and Fructus Ligustri Lucidi may be the result of cooperative action among multiple compounds with their multiple components working together to common affect. The exact roles of the different active components when used for treatment of osteoporosis remain to be further investigated.

## Conclusions

The combination of extracts from Herba Epimedii and Fructus Ligustri Lucidi demonstrated potential as a protective treatment for retinoic acid-induced osteoporosis in rats. The mechanism controlling the anti-osteoporosis effects of the combined extracts may involve the synergistic inhibitory effects on bone formation and bone resorption, thereby reducing the high turnover rate of osteoporotic bone metabolism. In addition, the combined extracts may be able to enhance the bone biomechanical properties and increase the bone mineral content.

## Abbreviations

TCM: Traditional Chinese medicine
BMD: Bone mineral density
TFE: Total flavonoids of Herba Epimedii
TIFL: Total iridoid and flavonoids of Fructus Ligustri Lucidi
ALP: Alkaline phosphatase
TRACP: Tartrate-resistant acid phosphatase
PICP: Procollagen type I carboxy-terminal propeptide
PINP: Procollagen type I amino-terminal propeptide
CTX: Carboxy-terminal cross linked telopeptide of type I collagen
